# Assessment of the N- and P-Fertilization Effect of Black Soldier Fly (Diptera: Stratiomyidae) By-Products on Maize

**DOI:** 10.1093/jisesa/ieaa089

**Published:** 2020-09-22

**Authors:** Daniel Gärttling, Sascha M Kirchner, Hannes Schulz

**Affiliations:** 1 Organic Farming and Cropping Systems, University of Kassel, Witzenhausen, Germany; 2 Section of Agricultural and Biosystems Engineering, University of Kassel, Witzenhausen, Germany; 3 Kuratorium für Technik und Bauwesen in der Landwirtschaft e.V. (KTBL), Darmstadt, Germany

**Keywords:** frass, plant, nutrition, residue, corn

## Abstract

To meet the growing demand for an alternative animal protein source, the Black Soldier Fly (BSF) *(Hermetia illucens)* industry is expanding. Thus, the valuation of its byproducts, foremost BSF frass, is getting more economic and ecological weight. Three different residues, BSF frass, larval skins, and dead adult flies, were compared with a mineral and an organic commercial fertilizer in a pot trial with maize (Zea mays L., [Poales: Poaceae]). byproducts were applied in three nutrient-based application rates (180; 215 kg N/ha; 75 kg P_2_O_5_/ha), and plant nutrients, physiological and yield parameters were measured at harvest date. Ground flies had the highest N-fertilizing effect of all byproducts, similar to commercial mineral and organic fertilizers used as controls, whereas its proportion of the BSF production systems’ output is low. Frass as the abundant byproduct showed comparably low N-fertilization effects. Its low N availability was attributed to volatilization losses, mainly driven by high pH and ammonium contents. BSF frass as the main byproduct output is more suited as a basic fertilizer or potting substrate amendment than as a short-term organic fertilizer. Postprocessing of frass seems reasonable. For a profound assessment of frass as fertilizer, several aspects (e.g., the overall impact of postprocessing, plant strengthening and plant protection potential, effects on microbial processes) must be clarified.

Aquaculture industry more than doubled its production since the year 2000 and provides nearly 50 % of the fish globally consumed nowadays (FAO 2018). In contrast, fishmeal and fish oil production decreased by 50% between 1994 and 2016 due to overfishing (FAO 2018). As 73 % of fishmeal and 71 % of fish oil produced is used for fish feed in aquaculture (FAO 2018), the demand for an alternative protein source is immense.

Black Soldier Fly (BSF) *(Hermetia illucens*) is a promising substitute for fishmeal. Depending on the fish species, between 25 and 100% of the fishmeal utilized in aquaculture can be replaced with BSF larvae meal (Belghit et al. 2019); promising studies have also been conducted on poultry, pigs and crustaceans. The protein-rich larvae of this insect can be reared on many substrates, e.g., food waste (Pastor et al. 2015) or even manure (Sheppard et al. 1994, Diener et al. 2015). As insect production companies put much effort in the automation of the processes, BSF rearing will become more cost-effective, especially in developed countries. A massive growth of the production capacities in temperate climates is to be expected (Derrien and Boccuni 2018).

With the increasing importance of the insect production sector, its often-promoted ecological sustainability needs to be assessed as an entity (Berggren et al. 2019), thereby contributing to a basis for decision making in politics and the industry itself. Unfortunately, there is a lack of knowledge about many aspects of insect production. One area that needs deeper research is the use of frass as potential soil amendment, regarding its effects on plant growth, its nutrient composition and its influencing factors on variation, and the ecological aspects of its application (Berggren et al. 2019). When agricultural sustainability and value-added environment-friendly application in agriculture or horticulture are explored, it is of essential importance to determine the actual fertilizing effect of frass.

European insect producers are mostly small or medium-sized companies, of which about 80% are currently rearing BSF; most of them for the use in pet food and aquaculture (Derrien and Boccuni 2018). Present production sums up to a few thousand tonnes per year, but production will expand substantially in the coming years (Derrien and Boccuni 2018). Frass will emerge toward a considerable byproduct as the industry grows rapidly; it represents 80–95% of the total output of a BSF production system (Devic 2016).

So far, only a few plant nutrition studies with BSF frass have been carried out, and its fertilizing effects remain unclear (Berggren et al. 2019). Choi et al. (2009) tested BSF frass against a commercial fertilizer on plots of cabbage, with no differences in growth and volume increase after 4 wk. In spring onions, BSF frass from two different rearing substrates improved yields to the same extent as the inorganic control (Devic 2016). Frass from BSF reared on pig slurry increased plant growth for basil and Sudan grass in a pot trial(Newton et al. 2005). Temple et al. (2013) compared a merchantable BSF frass fertilizer with worm compost and poultry manure in incubation, starter, and field trials using four experimental crops (potato, lettuce, Chinese cabbage, green bean). Frass had high N, P, and K concentrations and high availability of these, revealing its relative superiority to poultry manure and compost by the significantly strongest positive yield response.

However, high frass application rates can lead to growth inhibition (Newton et al. 2005, Temple et al. 2013, Alattar et al. 2016) and yield reduction (Newton et al. 2005, Temple et al. 2013), which often is attributed to NH_4_^+^-N toxicity (Temple et al. 2013, Alattar et al. 2016). This could be a reproducible phenomenon at high application rates of BSF frass. Negative effects of frass on plant growth and germination were found for other insect species as well (Silander et al. 1983, Kagata and Ohgushi 2012) but were associated to allelopathic effects.

The feeding substrate influences frass in its microbial composition as well as in its nutrient content (Poveda et al. 2019). Nitrogen is mainly excreted as urea, uric acid, and allantoin (O’Donnell and Donini 2017). The nitrogen fraction in the frass of terrestrial insects contains 9–27% of ammonium (Kagata and Ohgushi 2011), but most insects mainly excrete uric acid (Cochran 1985, Halloran et al. 2018). Allantoin breaks down into urea which then is converted into NH_4_^+^ by urease, leading to a high ammonium content in frass (Green and Popa 2012). The conversion of the mentioned nitrogen compounds of frass to ammonia should be relatively low due to its low moisture (Halloran et al. 2018). Although it is likely that ammonia emissions will increase if the moisture content of frass rises, e.g., due to changes in the production system or during fertilizer application, no measurements have been conducted yet (Green and Popa 2012, Halloran et al. 2018).

BSF frass has a higher pH value than most comparable fertilizers. Ma et al. (2018) stated that the maximum growth performance of BSF larvae can be reached on substrates with pH 6.0 or higher, while the larvae adjusted the substrate pH in these treatments to values from 8.0 to 8.5. A similar pH range from 8 to 9 was found by Green and Popa (2012) and Alattar (2012). Gärttling and Schulz (2019) reported a mean pH of 7.75 when averaging 14 frass analyses from different sources. The pH shift during the processing of the feeding substrate, which typically carries lower pH values, can be explained through 1) the incorporation of organic acids by the larvae and 2) rising NH_4_^+^ contents due to the decomposition of organic nitrogen compounds such as uric acid and allantoin in the frass (Green and Popa 2012).

Insect frass often contains chitin, which is known to have several beneficial effects on plant growth and health (Hadwiger 2013, Sharp 2013, Debode et al. 2016, Quilliam et al. 2020), functions as a nematicide and fungicide, and promotes mycorrhization, but in some cases appears to be phytotoxic (Sharp 2013). Also, BSF frass is reported to have insecticidal (Vickerson et al. 2013) and insect-deterring properties (Bradley and Sheppard 1984, Vickerson et al. 2013).

In nutrient analyses of BSF frass, it was classified as compound NPK fertilizer with 3.4% N, 2.9% P_2_O_5_, and 3.5% K_2_O on average and a neutral to alkaline pH (Gärttling and Schulz 2019). Frass fertilizers are, inter alia, marketed for organic production in the United States (OMRI 2019), in South Africa (SABS 2016), China (Halloran et al. 2016), and the European Union (FiBL 2018).

In this study, three different residues, BSF frass, larval skins, and dead adult flies, were compared with a mineral and an organic commercial fertilizer in a pot trial with maize. The effects of the mentioned fertilizers on plant growth and development as well as nutrient content and utilization were investigated. It is hypothesized that the BSF byproducts are comparable to organic commercial fertilizers in their plant-nutritional properties. This should be indicated by 1) similar nitrogen nutrition states, expressed in parameters like the nitrogen use efficiency (NUE) and the nitrogen nutrition index (NNI), 2) a similar habitus, which is described through leaf area and shoot:root ratio (SRR), and 3) a similar physiological age, which is related to dry matter (DM) content. Lastly 4), comparable fertilizers would result in similar yields (fresh and dry mass).

## Materials and Methods

### Fertilizer Sampling

Three byproducts of the BSF production were examined in the form in which they arise in the production plant. Frass (FR) samples were taken during the sieving process. The larval skins (LS) were separated from the larvae by winnowing. Adult flies (AD) were frozen and emptied directly into lockable buckets. A fraction of each material used for the fertilization experiment (unprocessed frass, unprocessed larval skins, frozen flies) was examined for quality parameters (pH (0.01 M CaCl_2_), dry mass, organic matter, residue on ignition—N, NH_4_^+^-N, P_2_O_5_, K_2_O, CaO, MgO, Na, S—microbial load (*Enterobacteriaceae, Salmonella*) (Supp Table 1 [online only]).

To assess fresh matter (FM) and organic matter (OM) obtained, insect substrates were dried at a low temperature of 39°C in order to minimize ammonia emissions. Larval skins were ground with a hammer mill at a sieve width of 0.5 mm, adult flies at 1.2 mm. The control fertilizers (granules) and the insect frass (powdery substance) retained their original grain size.

### Experiment Set-up

In the pot trial, the three fertilizers were applied each in three levels of 180 and 215 kg N/ha as well as 75 kg P_2_O_5_/ha, which was based on a soil surface area of 225 cm^2^ (15 × 15 cm) per pot. The variation of nontargeted nutrients due to different nutrient profiles was not adjusted, leading to application rates displayed in Table 2. Two control treatments were set up on the level of 180 kg N/ha. All treatments were replicated four times. For the organic-fertilized control (ORG), Phytogrieß GOLD and Provita Haarmehl-Pellets were mixed in the ratio 7:1. The mineral-fertilized control (NPK) contained commercial NPK fertilizer ‘COMPO Rosen Langzeit-Dünger’ (COMPO GmbH, Muenster, Germany). Because of their advantageous N:P ratios (Table 1), the controls also were comparable to the P-fertilized treatments (Table 2). The weighed fertilizer quantities were between 2.79 g (NPK, 180 kg N/ha) and 16.37 g (FR, 215 kg N/ha) per pot, corresponding to a range of 1.24–7.28 t/ha.

**Table 1.** Nutrient contents in fresh mass of the organic and mineral fertilizers used for the control treatments (information derived from data sheets)

	Fertilizer name	*N*	P_2_O_5_	K_2_O
1	COMPO Rosen Langzeit-Dünger (COMPO GmbH)	14.5	7	15
2	Provita Haarmehl-Pellets (Beckmann & Brehm GmbH)	14.1	0.89	0.24
3	Phytogries GOLD (Beckmann & Brehm GmbH)	5.5	3	2
4	Mixture of organic fertilizers (2 and 3)	6.6	2.7	1.8

**Table 2.** Overview of the experimental design of the fertilizer trial and the applied amounts of N, P_2_O_5_, and K_2_O

Applied fertilizer	Application level	N (kg/ha)	P_2_O_5_ (kg/ha)	K_2_O (kg/ha)
FR	N_max_	215	222	156
	N_uc_	180	185	131
	P_opt_	73	75	53
LS	N_max_	215	125	112
	N_uc_	180	105	94
	P_opt_	129	75	67
AD	N_max_	215	46	21
	N_uc_	180	38	18
	P_opt_	353	75	35
ORG	N_uc_	180	75	49
NPK	N_uc_	180	87	186

Among the applied fertilizers, FR is frass, LS is larval skins, AD is adult flies, ORG is organic mixed-fertilizer and NPK is mineral fertilizer. For the application levels, N_max_ is equivalent to 215 kg N/ha, N_uc_ to 180 kg N/ha and P_opt_ to 75 kg P_2_O_5_/ha.

For the experiment, a total of 44 plant pots with 15 × 15 × 22-cm dimensions were filled with 1.5 kg of preformulated low-nutrient potting soil (‘F.-E. Typ Nullerde’, Industrie-Erdenwerk Archut GmbH & Co. KG, Sauensiek, Germany) for datasheet see Supp Table 2 [online only]). The fertilizers were mixed into the upper 350 g of the potting substrate. Maize seed of the variety ‘PM PRALINIA’ (Planterra, BayWa AG, München, Germany) was sterilized on the day of sowing. For this purpose, the seeds were soaked for two and a half minutes in 10% hydrogen peroxide (H_2_O_2_), then rinsed three times with water and poured off. They were then immersed in 70% ethanol for 5 min and again rinsed three times. Floating seeds were removed. Four seeds per pot were sown at 5-cm depth, and plants were reduced to three after 1 wk.

The pots were watered with demineralized water to their water-holding capacity and readjusted gravimetrically. Target weights were elevated during plant growth to ensure the complete moistening of the pot volume. Those minimum and maximum pot weights were elevated during plant growth, so that the amount of water was enough to moisten the pots to the ground. Weeds were removed. In regular intervals, the pots were randomized using the randomization software Research Randomizer (Urbaniak and Plous 2013).

The average temperature in the greenhouse chamber was 20.9°C [coefficient of variation (CV) = 0.104], with 22.2°C (CV = 0.094) daytime and 19.3°C (CV = 0.048) nighttime temperatures (12: 12 h) (Fig. 2). Average relative humidity (RH) was 67.8 % RH (CV = 0.103), 68.0 % RH (CV = 0.061) at day, and 67.5 % RH (CV = 0.116) at night (Fig. 3). Sodium-vapor lamp lighting was set to 16 h a day.

**Fig. 1.**
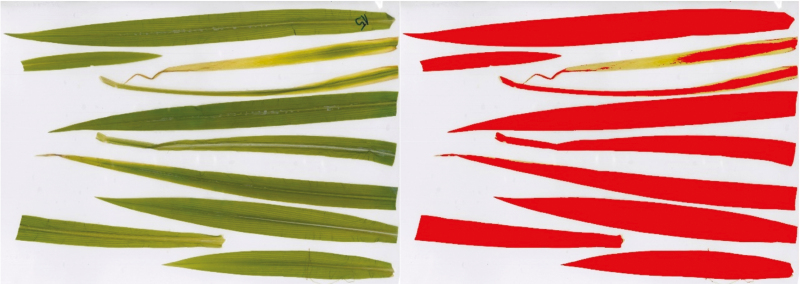
Original, scanned leaf image (left), and image after using the color threshold (right). Every colored area was selected separately for leaf area measurement, so irregularly colored parts (e.g., the leaf tip of the third leaf) could be excluded.

**Fig. 2.**
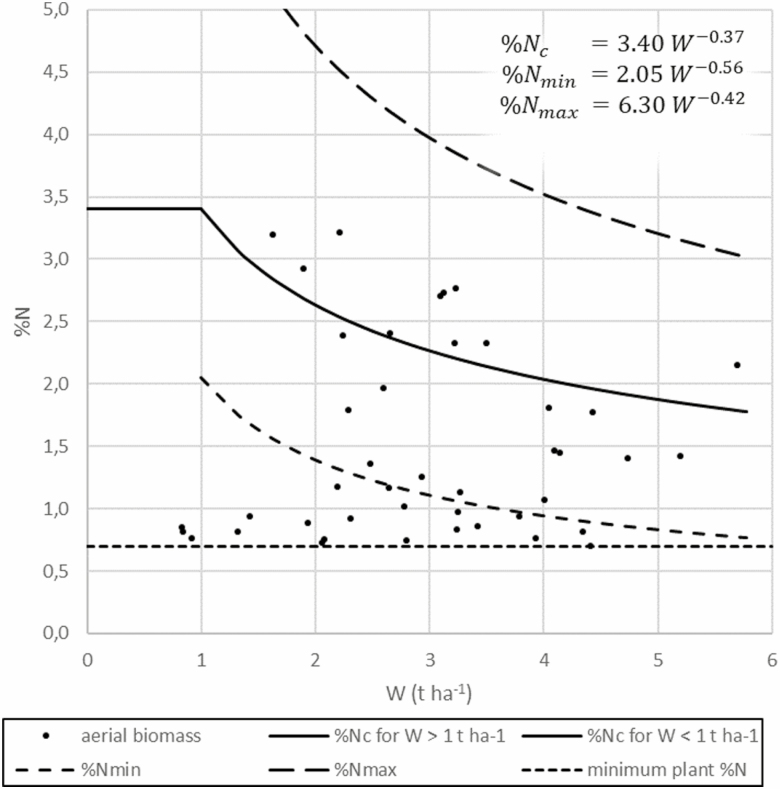
Time course of the mean temperature in the climate chamber during the day. Measurements took place every 12 min during the 10-wk experiment. Of each measurement time, mean and variation range (maximum – minimum) are depicted.

**Fig. 3.**
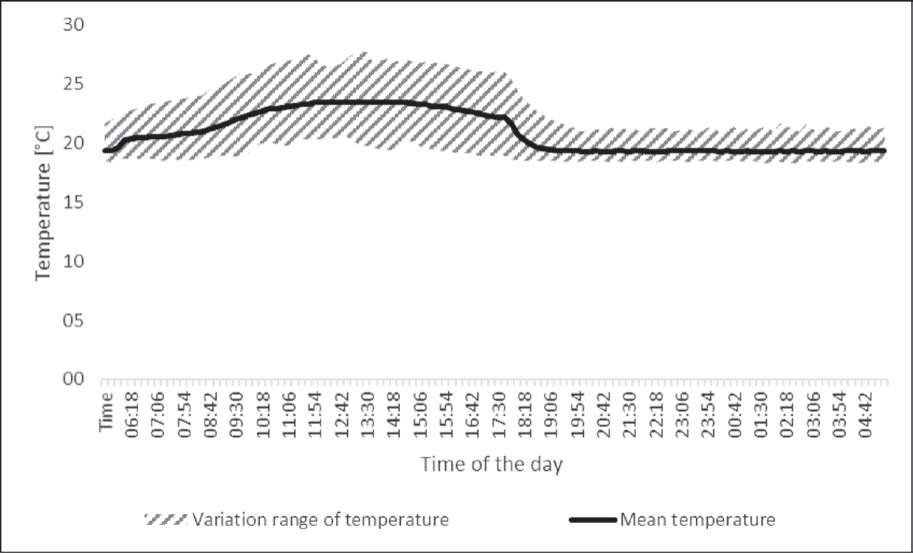
Time course of the mean relative humidity in the climate chamber during the day. Measurements took place every 12 min during the 10-wk experiment. Of each measurement time, mean and variation range (maximum – minimum) are depicted.

### Sampling and Data Collection

The experiment was completed after 73 d. The maize plants were cut off at the substrate surface and the entire aboveground biomass of a pot was pooled in a weighed aluminum dish. After determining the fresh weights, one representative plant per tray was selected and defoliated. The leaves were laminated in DIN A3 laminating pouches and scanned. The scanned lamination pouches were saved as images (JPEG, 8264 × 5840 pixel, 500 dpi). Leaf area was determined using the image analysis platform Fiji (Schindelin et al. 2012). After setting a known length (scan width or scanned ruler) as a scale, the living leaf area was separated by color threshold in a range from 165 to 215 in brightness and measured (for an example see Fig. 1). Doubled leaf areas were considered by polygon selection and added to the leaf area sum of the plant.

The remaining plants of the pot were weighed again, dried at 80°C to constant weight, and shoot dry mass content was determined to calculate DM yields from the fresh mass. Within the next 2 d, the roots were separated, washed, and dried in order to determine its dry weight. The shoots were then ground with a centrifugal mill, whereas the roots were not analyzed further.

For total carbon (C_t_) and total nitrogen (N_t_) analysis, 150 mg of plant powder were weighed in probation vials and analyzed via elemental analysis using the macro analyzer ‘vario MAX CHN’ (Elementar Analysensysteme GmbH, Langenselbold, Germany). For P analysis, two samples of ~0.5 g of plant powder were taken from each replicate to be reduced to ashes in the furnace (550°C for 10 h). The P analysis was carried out using method A 2.4.2.1 from Thun and Hoffmann (1991). Because of partially high P contents, the amount of filtrate used for inking was reduced to 0.5 ml.

### Nitrogen Nutrition Parameters

The instantaneous NNI was calculated as the ratio of current N concentration in aboveground biomass and the critical N concentration (%N_c_) for present yields (Lemaire 1997). Critical N concentration was calculated based on its exponential decay model equation on DM yields, as elaborated for maize by Lemaire [1997; %N_c_ = 3.40 W^−0.37^, where W is DM yield (in t/ha) of aboveground biomass].

The NUE was computed as the ratio of N output through N in aerial biomass and N input through fertilizer application (EUNEP 2015). As it should not be interpreted without considering N output and surplus (EUNEP 2015), these were calculated as DM yield per N_t_ in DM, respectively, the difference of applied N and this value (Lemaire 1997). The shoot:root ratio was obtained from the dry weights of each plant part. The C:N and the N:P ratio are based on the elementary nutrient forms.

### Data Processing and Statistical Analysis

Individual datasets were created for the time-related measures during growth, for the final analyses and for the final Yara-N and N_t_ results used for the regression function. A one-way analysis of variance (ANOVA) was carried out for all parameters measured at harvest date, followed by the appropriate post-hoc-test if the *P*-value indicated significant differences between the factor levels. Before applying the ANOVA, all parameters were tested on the assumptions of normality and homoscedasticity. To avoid distortion through extreme values, these were identified in a boxplot of the residues (>3 × height of the box) and excluded if they also showed a Cook’s Distance >1. Outliers were not excluded in order not to unbalance the dataset. The assumption of independency of the samples was assured by the experimental design. For normality, the Shapiro–Wilk test was carried out on the residues and for homoscedasticity, a Levene test was performed. When the assumptions were not met, a square-root- or ln-transformation was performed to fulfil the ANOVA assumptions. If this was successful, the means and the CIs were backtransformed to be displayed in the results after the ANOVA and Tukey’s post-hoc test had been carried out, if not, a Kruskal–Wallis test including the results of the pairwise comparisons was applied on the untransformed data. All statistical analysis was carried out with the SPSS ver. 24.0 (IBM Corp. 2016).

## Results

### Byproduct Analysis

In Table 3, the results of the analysis of the different insect fertilizers are displayed. *Salmonella* was not detected in any of the samples (data not shown), while *Enterobacteriaceae* was present in all of them (>150,000 CFU/g). Drying led to a reduction, whereas freezing did not. The pH value was relatively high in all insect-derived products (mean 7.75). The adult flies (AD) showed a higher water content (41.7 %), in consequence less FM and OM (55.7 %) contents and residue on ignition (2.6 %) than the frass (FR) and the larval skins (LS).

**Table 3.** Nutrient contents of the BSF by-products

Parameter	Unit	Frass	Larval skins	Flies
Physical and technical parameters				
pH		9.0	7.4	6.9
Water content	% DM	18.3	15.5	71.5
Organic matter	% DM	90.4	91.2	95.5
Residue on ignition (550°C)	% DM	9.6	8.8	4.4
Value-determining components				
Total nitrogen (N_t_)	% DM	3.30	4.80	11.30
Ammonium nitrogen (NH_4_^+^-N)	% DM	1.00	0.23	0.74
Phosphate (P_2_O_5_)	% DM	3.40	2.80	2.40
Total potassium (K_2_O)	% DM	2.40	2.50	1.10
Calcium (CaO)	% DM	0.40	0.70	<0.1
Magnesium (MgO)	% DM	1.00	0.68	0.48
Total sulfur (S)	% DM	0.53	0.48	0.57
Sodium (Na)	% DM	0.26	0.22	<0.17

The analytical methods used are displayed in Supp Table 1 (online only).

Although the N_t_ content in FM and DM were the highest in AD, the most ammonium (NH_4_^+^-N) was found in FR (Table 3). N:P ratios were 2.22 for FR, 3.93 for LS, and 10.79 for AD. The K_2_O contents were lower than N and P_2_O_5_ in all insect-related substrates. However, when considering the element forms, potassium concentrations were lowest in AD, whereas phosphorus concentrations were lowest in FR and LS.

### Plant Analyses of Maize

Maize nutrient and plant growth parameters of control treatments (ORG, NPK) were comparable to each other (Table 4; *P* > 0.05). Generally, DM contents decreased with increasing N supply, but significant differences were only found between the highest-yielding (NPK control, 10.9%) and the lowest-yielding (FR P_opt_, 14.3% and LS P_opt_, 15.0 %) treatments. The N_t_ content tended to increase with higher N fertilization. P_2_O_5_ was high, where N_t_ was low and vice versa. Since C_t_ did not show notable differences, the plant C:N ratio was mainly influenced by N_t_. The N:P ratio increased in the order FR < LS < controls < AD and, within one fertilizer, in the order P_opt_ < N_uc_ < N_max_. At the N_max_ application level, AD fertilization led to higher N_t_ and lower P_2_O_5_ contents than FR and LS treatments, leading to a wider plant N:P ratio. Due to differing N_t_ contents, the plant C:N ratio was also lower for AD N_max_, but not significantly.

**Table 4.** Nutrients, fractions, and nutrient ratios of the aerial maize biomass

Fert.	Appl. level	DM (% FM)	N_t_ (% DM)	P_2_O_5_ (% DM)	C:N ratio	N:P ratio
FR	N_max_	12.705 (±0.518)^ab^	1.06, [0.761, 1.476]^ab^	2.04, [1.404, 2.964]^d^	41.051 (±4.228)^bcd^	1.19, [1.023, 1.385]^bc^
	N_uc_	13.966 (±0.661)^ab^	0.934, [0.662, 1.319]^ab^	2.149, [1.162, 3.974]^d^	46.477 (±5.543)^cd^	0.997, [0.669, 1.485]^ab^
	P_opt_	14.317 (±0.119)^b^	0.809, [0.753, 0.869]^a^	2.568, [1.801, 3.66]^d^	51.047 (±1.027)^d^	0.722, [0.498, 1.047]^a^
LS	N_max_	12.879 (±0.961)^ab^	1.147, [0.661, 1.99]^abc^	1.624, [0.851, 3.101]^cd^	39.595 (±6.51)^bcd^	1.618, [1.377, 1.9]^c^
	N_uc_	14.645 (±0.767)^ab^	0.869, [0.623, 1.214]^ab^	1.288, [0.942, 1.761]^cd^	50.496 (±4.992)^d^	1.545, [1.037, 2.301]^c^
	P_opt_	15.018 (±0.269)^b^	0.823, [0.676, 1.001]^a^	1.43, [1.062, 1.927]^cd^	51.869 (±3.436)^d^	1.319, [1.158, 1.503]^bc^
AD	N_max_	12.887 (±0.758)^ab^	2.153, [1.369, 3.388]^d^	0.377, [0.228, 0.624]^ab^	21.159 (±3.487)^ab^	13.092, [12.019, 14.261]^e^
	N_uc_	13.185 (±0.877)^ab^	1.943, [1.084, 3.48]^cd^	0.343, [0.15, 0.784]^a^	23.923 (±4.403)^ab^	12.975, [9.874, 17.05]^e^
	P_opt_	11.815 (±0.488)^ab^	2.787, [2.061, 3.77]^d^	0.766, [0.393, 1.494]^bc^	15.868 (±1.81)^a^	8.339, [5.764, 12.066]^d^
ORG	N_uc_	13.417 (±0.748)^ab^	1.537, [0.999, 2.367]^bcd^	0.438, [0.257, 0.746]^ab^	29.932 (±4.242)^abc^	8.045, [6.66, 9.717]^d^
NPK	N_uc_	10.902 (±0.572)^a^	2.052, [1.356, 3.107]^cd^	0.804, [0.589, 1.098]^bc^	21.866 (±2.967)^ab^	5.853, [4.976, 6.885]^d^
*F*-value		*F*(10,33) = 3.435	*F*(10,33) = 13.181	*F*(10,33) = 19.619	*F*(10,33) = 10.654	*F*(10,33) = 171.858
*P*-value		*P* = 0.004	*P* = 0.000	*P* = 0.000	*P* = 0.000	*P* = 0.000

Ln-transformed and backtransformed data are means with confidence intervals (CI95 = []), unprocessed data are means (± SE), *n* = 4; α = 0.05.

A similar pattern appeared when the plants were fertilized at N_uc_ level, resulting in a grouping of FR/LS versus AD/controls: N_t_ contents of FR and LS were comparable inter alia and lower than AD, which was comparable to the NPK control. ORG was in between and not significantly different from any other treatment. P_2_O_5_ contents were low for AD and the ORG control and high for LS and FR, leaving the NPK control in between. This ranking followed the P_2_O_5_ application rates in this treatment. The C:N ratio of AD was lower than that of FR and LS, which were comparable to each other. The plants’ N:P ratio decreased in the order AD > ORG > NPK > LS > FR, analogous to the fertilizers’ N:P ratios.

In the P_opt_ treatment, the N_t_ contents followed N application rates. Despite an equal P_2_O_5_ application rate, P_2_O_5_ contents were high in lower-yielding FR and LS and low in higher-yielding AD and ORG.

### Nitrogen Nutrition of Treatments

Concerning the NUE, N output and NNI in the moderately fertilized N_uc_ treatments, FR and LS grouped together, as well as AD and the controls (Table 5). This difference between FR/LS and AD was also visible in the N_max_ application rate. Within all fertilizers, NUE increased with higher N application rates except for the highly N fertilized AD P_opt_ treatment. In this treatment, the highest NNI (1.20) and N surplus (270.32 kg/ha) was observed.

**Table 5.** Nitrogen nutrition key figures of the fertilizing experiment

Fertilizer	Appl. level	NUE (%)	N output (kg/ha)	N surplus (kg/ha)	NNI
Frass	N_max_	14.96, [12.15, 18.42]^abc^	32.169, [26.122, 39.616]^c^	182.629 (± 2.07)^cd^	0.47, [0.358, 0.617]^c^
	N_uc_	15.26, [11.55, 20.16]^abc^	27.467, [20.787, 36.294]^c^	152.231 (± 2.247)^abcd^	0.409, [0.322, 0.52]^bc^
	P_opt_	10.6, [7.48, 15.02]^a^	7.745, [5.466, 10.973]^a^	65.107 (± 0.952)^a^	0.244, [0.218, 0.273]^a^
Larval skins	N_max_	17.29, [15.54, 19.24]^bc^	37.151, [33.38, 41.349]^cd^	177.773 (± 1.286)^bcd^	0.521, [0.354, 0.766]^cd^
	N_uc_	14.54, [11.12, 19.02]^abc^	26.18, [20.015, 34.243]^c^	153.54 (± 2.115)^abcd^	0.384, [0.302, 0.49]^bc^
	P_opt_	11.8, [10.08, 13.82]^ab^	15.226, [13.001, 17.831]^b^	113.724 (± 0.751)^abc^	0.304, [0.269, 0.344]^ab^
Adult flies	N_max_	34.3, [28.72, 40.96]^de^	73.774, [61.78, 88.095]^e^	140.905 (± 4.078)^abcd^	0.999, [0.738, 1.351]^ef^
	N_uc_	32.53, [27.91, 37.91]^de^	58.557, [50.245, 68.244]^e^	121.219 (± 2.874)^abcd^	0.859, [0.624, 1.183]^ef^
	P_opt_	22.34, [12.65, 39.43]^cd^	78.807, [44.643, 139.115]^e^	270.321 (± 14.873)^d^	1.204, [1.112, 1.304]^f^
Organic fertilizer	N_uc_	30.94, [21.51, 44.51]^de^	55.701, [38.724, 80.121]^de^	123.218 (± 6.509)^abcd^	0.728, [0.502, 1.054]^de^
NPK	N_uc_	46.95, [42.33, 52.09]^e^	84.521, [76.192, 93.761]^e^	95.307 (± 2.748)^ab^	1.019, [0.78, 1.332]^ef^
*F*-value		*F* (10,33) = 30.867	*F*(10,33) = 71.209	*F*(10,33) = 102.661	*F*(10,33) = 41.167
*P*-value		*P* = 0.000	*P* = 0.000	*P* = 0.000	*P* = 0.000

Ln-transformed and backtransformed data are means with confidence intervals (CI_95_ = []), unprocessed data are means (±SE), *n* = 4; α = 0.05.

**Fig. 4.**
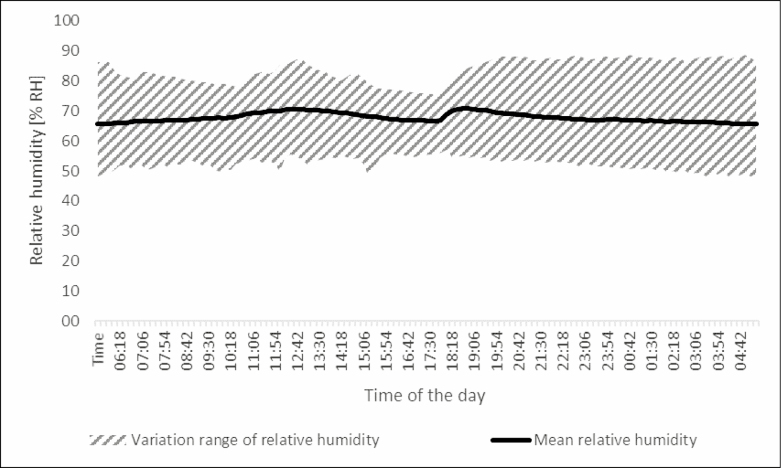
Relationship between %N and aerial biomass (W). %Nc function with minimum (%Nmin) and maximum (%Nmax) envelope curves from Lemaire (1997). Constant minimum plant N based on the least %N of the dataset.

Aboveground DM yields of maize and their corresponding nitrogen concentration (%N) are shown in Fig. 4. They are put in context to the critical N concentration curve for maize and its minimal and maximal envelope curves published by Lemaire et al. (1997). Since Lemaire’s equations for N_c_% are only valid for yields >1 t DM/ha, a constant of 3.40% was used for %N_c_ for 3 of the 44 pots, thereby following his proposal. The observed minimum plant %N was at 0.698% DM. There is a greater part of the values below the %N_c_ curve than above. Even about half of the pots (*n* = 20) were below Lemaire’s minimal envelope curve and allocated near the minimum plant %N constant. When reversed, the displayed values in Fig. 4 build up an optimum curve of the DM yields against %N, having the highest scatter in the optimum range. A very similar graph can be observed when %N is replaced by the NNI.

### Yield-Related Plant Parameters

At the same N nutrition level, the applied fertilizers were not distinguishable from each other in these parameters (Table 6). In leaf area, the NPK treatment was comparable to AD and ORG. The increase in N supply from N_uc_ to N_max_ was not reflected in growth parameters, but N supply below N_uc_ revealed negative effects. Control and AD treatments were not distinguishable from each other.

**Table 6.** Growth parameters at harvest date

Fertilizer	Appl. level	Yield_aereal_ [g DM]	Shoot:root ratio	Leaf area [cm^2^]	Final plant height [cm]
Frass	N_max_	6.828, [5.498, 8.479]^bc^	9.314 (±1.022)^ab^	506.565, [396.805, 629.711]^bc^	95.4 (±2.9)^abc^
	N_uc_	6.613, [4.229, 10.341]^bc^	10.525 (±0.679)^abc^	521.757, [402.49, 656.479]^bc^	96.7 (±5.1)^abc^
	P_opt_	2.153, [1.519, 3.052]^a^	6.918 (±0.477)^a^	138.345, [49.737, 271.311]^a^	74.5 (±3.9)^a^
Larval skins	N_max_	7.294, [4.648, 11.444]^bc^	12.832 (±1.318)^bc^	590.879, [467.133, 729.148]^c^	102.1 (±2.4)^bc^
	N_uc_	6.78, [4.531, 10.145]^bc^	12.085 (±0.71)^bc^	512.479, [379.714, 665.112]^bc^	101.2 (±3.1)^bc^
	P_opt_	4.162, [3.141, 5.515]^ab^	10.628 (±0.783)^abc^	309.936, [249.957, 376.359]^b^	80.1 (±2.0)^ab^
Adult flies	N_max_	7.706, [4.856, 12.228]^bc^	12.846 (±0.566)^bc^	740.221, [616.78, 874.914]^cd^	106.5 (±3.3)^c^
	N_uc_	6.787, [3.308, 13.926]^bc^	10.645 (±0.544)^abc^	654.541, [521.901, 802.184]^cd^	98.1 (±8.9)^bc^
	P_opt_	6.36, [2.703, 14.963]^bc^	12.944 (±0.436)^bc^	662.496, [439.752, 930.722]^cd^	99.8 (±7.5)^bc^
Organic fertilizer	N_uc_	8.15, [5.724, 11.604]^bc^	13.44 (±0.718)^c^	622.852, [363.09, 952.282]^cd^	103.4 (±1.3)^bc^
NPK	N_uc_	9.272, [6.11, 14.07]^c^	13.68 (±1.176)^c^	912.644, [627.904, 1250.471]^d^	108.3 (±5.7)^c^
*F*-value		(10,33) = 7.002	*F*(10,33) = 6.439	*F*(10,33) = 17.672	*F*(10,33) = 4.849
*P*-value		0.000	0.000	0.000	0.000

Ln-transformed and backtransformed data are means with confidence intervals (CI_95_ = []), unprocessed data are means (±SE), *n* = 4; α = 0.05.

Overall, growth parameters tended to increase with higher N application and showed better performance of the controls, especially NPK, over the insect-derived treatments. N application levels affected plant growth parameters more strongly than different N availability caused by the fertilizer’s characteristics; in that aspect, only trends were visible.

## Discussion

### Byproduct Characteristics

High DM contents of BSF frass improved its conservation properties and led to high nitrogen concentrations in FM (Table 3) compared with other farm manures and composts which (apart from chicken manure) are below 1% in FM (LfL Bayern 2018). Thus, lower application rates are needed for the nutrient application. An even N: P_2_O_5_ ratio with slightly lower K_2_O contents (Table 3) is not common among farm manures. In most manures, N dominates P_2_O_5_, as is the case for the nitrogen removal of most agricultural crops (LfL Bayern 2018). Although the NH_4_^+^-N fraction of N_t_ was very high for frass from terrestrial insects (Kagata and Ohgushi 2011), it was low compared with other farm manures (Gutser et al. 2005), and ammonia volatilization with the increasing humidity after application was possible due to the high pH observed (Table 3).

Both LS and AD fertilizers showed higher N_t_ but lower NH_4_^+^ concentrations than FR, combined with moderate pH values (Table 3). The fertilizer gained from adult flies has by far higher N concentrations in DM but should be dried to allow grinding and enhance conservation properties. Based on its N:P ratio, AD was the only byproduct fertilizer that was nitrogen dominated. AD had N concentrations comparable to usual commercial organic fertilizers.

Although growth depression after frass application occurred in other experiments, this was not the case for this experiment. Alattar et al (2016) observed it for sweet corn, which was associated with ammonium toxicity. Although corn was comparably insensitive to ammonium toxicity (Moritsugu et al. 1983, Spiegel et al. 1986), it affected sweet corn to a greater extent than silage maize (Hauck et al. 1984).

As Berggren et al. (2019) summarized, the current data situation regarding the effects of frass fertilization on plant growth is small and unclear in its outcomes. Different frass qualities critically influenced its effects on plant growth (Kagata and Ohgushi 2012). The authors stating negative effects of frass application on plant growth (Newton et al. 2005, Temple et al. 2013, Alattar 2016) compared it with compost or similar materials at fresh weight or volume equivalents. However, the utilized frass showed threefold higher %N in FM compared with common biowaste composts (LfL Bayern 2018), in addition to higher NH_4_^+^ concentrations. When lower rates, e.g., comparable to manure application, were applied, like in Zahn (2017), an optimum effect in plant growth was observed. Based on the reviewed literature, the characteristics of frass that lead to plant growth inhibition could not be identified definitively. The choice of silage maize as experimental crop and comparably low application rates may have helped to avoid negative effects on plant growth.

The finding that the concentration of *Salmonella* spp. was below the detection limit was concordant with the literature (Lalander et al. 2013, 2015), where an effective reduction of *Salmonella* spp. through BSF larvae was reported. *Escherichia coli* as a member of *Enterobacteriaceae* was described to be reduced in substrates through BSF treatment (Erickson et al. 2004, Liu et al. 2008). However, those suppressive effects were partly attributed to temperature (Liu et al. 2008) and uncharged ammonia concentrations (Lalander et al. 2015). The fact that high *Enterobacteriaceae* infestation was present in all BSF fertilizers could either have resulted from differences in the above-mentioned parameters or from a different proportion of *E. coli* in *Enterobacteriaceae* (Lalander et al. 2015). Drying (80°C, 1 h) was the better way for the reduction of *Enterobacteriaceae* in comparison to freezing but did not lead to an eradication. Both increased DM contents (Himathongkham and Riemann 1999) and high drying temperatures (Ghaly and Alhattab 2013) could have reduced the microbial load. However, drying manures of high pH at high temperatures can lead to a substantial volatilization of ammonia (Ghaly and Alhattab 2013, Pantelopoulos et al. 2016). The effects of different drying temperatures and durations were not tested.

### Nitrogen Fertilizing Effect Through Insect-Derived Fertilizers

High N supply led to low DM, which was most clearly visible for AD and NPK treatments (Table 4). Also treatments with high DM yields exhibited low DM contents. A well-nourished and therefore high yielding plant matures as fast as a plant in N deficiency (Marschner 2012) and, as a result, generative growth which is associated with higher DM contents is delayed. N nutrition above N_uc_ did not lead to significantly improved growth parameters (Table 6); thus, as predicted by Lemaire (1997), the N uptake optimum was surpassed for N_max_ treatments. Especially in the low-N-fertilized treatments, N was limiting and affected DM yields (and other growth parameters). The span of N application used for this experiment seemed great enough for displaying the optimum NUE (Table 5). In on-field cropping systems, the NUE was by far higher than the values determined in this experiment (EUNEP 2015). This was due to premature harvest, the presowing application of the full N rate which is unusual in maize cropping and the use of a soil that had bad N replacement characteristics.

The instantaneous NNI indicates the current nutrition status of the plant and is optimal when equal to 1. Oversupply is indicated above and N deficiency below this threshold; however, low discrepancies do not instantly affect yields. N_t_ application rates principally were high enough for the maize plants to reach an NNI around 1, but this was only the case when adult flies, organic or mineral fertilizer were applied (Table 5). Thus, nitrogen was less plant-available in FR and LS treatments and led to a malnutrition as the amounts of plant-available N was not high enough.

In this pot trial, adult fly fertilizer contributed better to N supply than larval skins and frass. This was reflected in various parameters linked to N nutrition. The low plant C:N ratios of AD were close to the values of the NPK control (Table 4), as no differences were visible in C_t_ contents but N_t_ was clearly higher in AD and the controls, and thereby indicated high N availability for these fertilizers (Griffith et al. 2000). There was no difference between the leaf area of AD and the control (Table 6), suggesting comparable N nutrition, since leaf area was affected positively by nitrogen (Chen et al. 2014). With increasing N deficiency, the ratio of metabolic plant mass to total biomass decreased (Lemaire 1997). As the metabolic plant mass was closely linked to the leaf mass (Lemaire 1997), a reduction of leaf area through N deficiency in FR and LS treatments was probable.

The NUE is expected to have an optimum curve with increasing N input, when no soil N is available (EUNEP 2015). Within FR or LS fertilized treatments, the NUE increased with N supply because N outputs increased faster than N surpluses but did not show an optimum curve, suggesting a low N supply of the fertilizers (Table 5). FR and LS showed a reduced NUE, achieving low N outputs and leaving high surpluses, and the NNI, which was far below 1 but increased with N application rates, indicated that the plants’ N supply was suboptimal.

Good N nutrition for AD, ORG, and NPK treatments was indicated by the NNI, which was around 1 for these fertilizers. These better-nourished plants showed a higher NUE with higher N outputs as well as lower N surpluses. N supply was excessive in AD P_opt_ (highest N surplus overall, NNI considerably above 1) and lowered the NUE compared to the lower-fertilized AD treatments. Although the N output still increased, overfertilization resulted in lower DM yields and leaf areas than in the moderately fertilized AD treatments.

N deficiency and overfertilization, for which %N is a reliable indicator (Lemaire 1997), both lower yields (Marschner 2012), leading to the described optimum curve for DM yields against %N (Fig. 4). Within one certain application level and N availability, higher yields decreased %N, which was assumed to be a natural phenomenon (Lemaire 1997) and contributed to the scatter around the optimum range. Many of the lower-fertilized plants were below Lemaire’s minimum envelope curve for %N, which means that a considerable amount of the pots must have suffered N deficiency. The minimum plant %N was observed to be 0.698% DM, thereby validating the minimum plant %N of 0.7% DM for maize proposed by Lemaire (1997).

In many parameters, the controls were on a par, but lower %N_t_ (Table 4) and instantaneous NNI (Table 5) indicated a slight disadvantage for the organically fertilized control. The SRR increased in the order FR < LS < AD (Table 6), which indicated an increasing plant N supply (Bonifas et al. 2005).

Generally, when comparing the means of the growth- and nutrition-related parameters for FR and LS in the same application level, LS largely surpassed FR. Most of the differences were explained with N nutrition, so frass-N must be considered less plant-available. This is interesting because frass had by far the highest proportion of NH_4_^+^-N in N_t_ (30% vs. 5%, Table 3), which commonly is estimated to be highly plant-available. AD fertilizer (6,3 % NH_4_^+^-N of N_t_) was not distinguishable from the controls when omitting the overfertilized AD P_opt_ treatment. Apparently, NH_4_^+^-N content was not a good indicator for plant-availability for the byproduct fertilizers in this experiment, either because other N fractions had greater influence on plant availability or because NH_4_^+^ volatilized as ammonia.

The direct uptake of organic nitrogen compounds is most common for urea and amino acids, but the extent of contribution to plant nutrition is still under investigation (Marschner 2012). Allantoin can, in principle, also be assimilated directly from the soil (Desimone 2002). The occurrence of these nitrogen fractions in frass of locust was reported in former publications (Cochran 1985, O’Donnell and Donini 2017) but was not investigated within this work.

### Phosphorus Fertilizing Effect Through Insect-Derived Fertilizers

The fertilizing effects regarding P could not be assessed isolated because the treatments comparable in P (P_opt_ and controls) carried different N application rates. Thus, the plant P concentrations must be interpreted in context to plant N. An indicator for a balanced nutrition of N and P is the N:P ratio of the aboveground biomass.

The sequence of the fertilizers in their N:P ratios was maintained in the plants’ N:P ratios. High fertilizer N:P ratios (AD, ORG, NPK) tended to widen when transferred into plant N:P ratios, low ratios (FR, LS) were narrowed (Tables 3 and 4). In field trials, the maize N:P ratio of aboveground biomass was around 5.75 (LfL Bayern 2018). The N:P ratios of ORG and especially NPK were closest to this value, so the balance of N and P nutrition was near the optimum. AD treatments had the widest plant N:P ratios and thereby seemed to exhibit the highest N supply (Ciampitti et al. 2013). At very high N application rates (AD P_opt_), the ratio decreased because P assimilation increased faster than N assimilation. Lowered yields (Table 6) and NUE (Table 5) indicated N oversupply when fertilizing on phosphorus demand, so the fertilizer’s N:P ratio did not meet the optimum assimilation ratio of maize.

The close fertilizer N:P ratio in FR and LS led to an even lower plant N:P ratio caused by high plant P concentrations (Table 4). Plants when experiencing N deficiency respond through the enhancement of root growth which results in high root DM weights and a lower SRR (Marschner 2012). As P is not as soluble as N, the enhanced root system improves more P than N nutrition (Blume et al. 2010), resulting in higher P concentrations in DM. Thus, the increased P concentrations of FR and LS treatments must be rather associated with N deficiency than with pure P fertilizing effects.

However, high NH_4_^+^ fertilization stimulates P assimilation (Marschner 2012) and could thereby have contributed to the higher P concentrations. Another reason for high P contents could be an increased mycorrhization (Grant et al. 2005), as reported by Zahn (2017) and Lovett and Ruesink (1995) in frass. However, no parameters were investigated in this experiment that could support this hypothesis.

### Discussion of Methods

The choice of maize as experimental crop was appropriate, as it demands the main part of N and P within the 10-wk growing period (Ciampitti et al. 2013) and is not affected severely by NH_4_^+^ toxicity (Moritsugu et al. 1983), which was an obstacle reported in former frass experiments (Temple et al. 2013, Zahn 2017).

By drying the insect-derived fertilizers before application, ammonia volatilization was expected and could have occurred. The proportions of NH_4_-N in total nitrogen (Table 3) were high compared with other BSF frass analyses (Gärttling and Schulz 2019) but still lower than poultry manure and various slurries (Gutser et al. 2005). Therefore, a tolerably low volatilization was expected, as drying was necessary to enhance conservation properties and as freeze-drying was not available. However, ammonia losses were not investigated and should be quantified in future to assess the volatilization risk of this process. Ammonia losses during postprocessing could partially explain the bad performance of frass as well as the absence of ammonium toxicity symptoms in the plants. As dry frass was associated with ammonia volatilization during rearing and storage (Halloran et al. 2016), the impact of the rearing system on the nutrient contents of frass should be addressed in future research.

The utilized experimental design with two nitrogen-balanced and one phosphorus-balanced application level without an additional N-leveling was not suitable to assess the phosphorus fertilizing effect of the fertilizers, as it was distorted by alternating N nutrition. A better but more complex design would have been a solely N-based increase series, maybe in combination with a separate P-increase series. However, N-leveling with a mineral fertilizer in this series is not appropriate to offset distortion by different N nutrition because of the fertilizers’ variable N mineralization characteristics.

The variation in yields at the same NNI indicated that the instantaneous NNI did not reflect the N supply during growth appropriately. Higher-yielding plants suffering from N deficiency at harvest date had a better N supply during growth than lower-yielding plants of the same NNI. For an increased informative value of the NNI, it could have been integrated over the growth period, thereby demanding a preharvest of additional pots. The data of the %N_c_ equations for maize were obtained from a series of field experiments. The main ecophysiological reason for the decrease of %N_c_ was suggested to be the changing ratio of structural and metabolic tissue (Lemaire 1997). Under greenhouse conditions, stem thickening was slower than it would have been expected in open field. With a slower increase of the fraction of structural tissue in plant DM, %N_c_ was estimated to be lower, maybe resulting in an overestimation of the NNI and, thus, the plant nutrition status.

### Application Purposes and Implications for Postprocessing

Although larval skins and especially adult flies exhibited better fertilizing effects than frass, a separate fertilizer processing would not be economical, as both byproducts are a quite small part of the byproduct output of BSF production. Thus, both substrates could be used to blend with the frass fertilizer, which is by far the greatest by-product output (Devic 2016), thereby improving the N:P profile of this substrate. The problems of different particle sizes of the products could be overcome by milling, sieving, and/or pelletizing. However, a further valuation by chitin extraction could also be possible (Cammack and Tomberlin 2017).

Currently, most of the frass fertilizers are marketed as garden fertilizers, but its use especially in organic agriculture and horticulture is also possible, as there is a higher demand for high-value organic fertilizers. Based on the performance in this trial, an application in basic fertilization is recommended, so it could be further supplemented by other fertilizers, oriented toward the needs of the crop. Slow-releasing N fertilizers also provided N to the subsequent crop, which ultimately increased their long-term efficiency (Gutser et al. 2005).

As the analyses on frass pH and NH_4_^+^ concentrations revealed favorable conditions for ammonia volatilization, postprocessing of the substrate seems reasonable but is not well-explored. Drying reduces phytotoxic effects (Alattar et al. 2016) and, as also shown in this study for *Enterobacteriaceae*, pathogens (Himathongkham and Riemann 1999, Ghaly and Alhattab 2013), but increases the risk of ammonia volatilization (Kagata and Ohgushi 2012, Halloran et al. 2016). Acidification of manures is known to reduce ammonia volatilization in farm manures (Pantelopoulos et al. 2016) and can also be used for chitin-rich substrates (Spiegel et al. 1986). Acidified manures show low ammonia volatilization even at high drying temperatures (Pantelopoulos et al. 2016), which could also improve hygienization effects. The amplification of the C: N ratio through blending with compost is suggested to reduce volatilization (Spiegel et al. 1986, Cortes Ortiz et al. 2016) and opens new application purposes, e.g., as formulated potting substrate. Postprocessing is not only recommendable to reduce greenhouse gas emissions of the whole production system but can contribute to improve the N fertilizing effect of frass to widen the application purposes in agriculture and horticulture.

### Conclusions

The hypothesis of nutritional qualities of all insect derived fertilizers being comparable with other organic fertilizers was not met. BSF frass itself performed worst in most parameters of the conducted trial, especially concerning its N fertilization effect. Plants fertilized with frass showed lower performance in NNI and NUE as well as smaller leaf area and narrower SRR, compared with adult fly fertilizer and especially the controls. Thus, nutrient status and habitus of frass and AD/controls were assumed to differ. DM contents indicating physiological age as well as yields displayed minor differences but seemed more related to application levels than to the choice of fertilizer, which may have resulted from the limited duration of the experiment and premature harvest. Due to the experimental design, the P-fertilization effects found for frass could not clearly be separated from the N-fertilization effects.

Although drying seems to be reasonable for conservation and hygienization purposes, it may have led to substantial volatilization of ammonia, thereby affecting the performance of frass in the trial. Other conservation methods or acidification prior to drying seem reasonable. Therefore, the results point to BSF frass use as a possible lower-level organic composite NPK fertilizer.

The literature on BSF frass suggests several beneficial and disadvantageous effects which have not been assessed in this study. For a valuation of frass as a fertilizer at larger scale, this seems to be a promising research area, e.g., regarding plant protection characteristics or for identifying and avoiding phytotoxic effects through frass application.

## Supplementary Material

ieaa089_suppl_Supplementary_Tables
