# Supplementary material for: Assessment of the N- and P-Fertilization Effect of Black Soldier Fly (Diptera: Stratiomyidae) By-Products on Maize
Source: J Insect Sci. 2020 Sep 22;20(5):8. doi: 10.1093/jisesa/ieaa089 (PMC7508297; doi:10.1093/jisesa/ieaa089)
Supplement: ieaa089_suppl_Supplementary_Tables [file ieaa089_suppl_supplementary_tables.docx]

[Excerpt from [Doc38439682-764496701](https://mc.manuscriptcentral.com/jis?DOWNLOAD=TRUE&PARAMS=xik_24EvWph1iYhABsgLF7cMtf564fuWtXu89Uo5osgYGJq9hBSXX9u1sjeoyESE8k8sAtfVY55NKuo3S1sQd4Wr9AkKSHmMCv2JJRdXqEma9Z6sWY6k6tCipVSmJ2CMhWyPS5bL7beBWxKbBbmYi9ntXM2hUhBcohx3BLpDhM6dNxn1ypWsUyL99TroE3NqzjMHe78Vn7QVoshm7mYUJvVuBDyDEVJNuSrEojdqizA7KeACYXgjGozfHA3oBtpp8Rc124FVXN6)]

**Supplementary material**

**Supp. Table S1: Analytical methods used for the analyses of the by-products of BSF production.**

| **Parameter** | **Analytical method** |
| --- | --- |
| Dry mass [%] | DIN 12880:2007-05 (DIN 2007) |
| Organic matter | VDLUFA II, 10.1:1999 (Bassler 1995) |
| Residue on ignition (550 °C) | VDLUFA II, 10.1:1999 (Bassler 1995) |
| pH | DIN 12176:1998-06 (CEN 1998) |
| N_t_ | Reg. (EC) 2003/2003, IV, 2.3.2:2003-10 |
| NH_4_^+^-N | Reg. (EC) 2003/2003, IV, 2.1:2003-10 |
| P_2_O_5_, K_2_O, MgO, Na_2_O, CaO, S, Cu, B, Mn, Zn, Fe | ISO 11885:2009-09 |
| *Enterobacteriaceae* | ISO 21528-2:2017-06 |
| *Salmonella spp.* | ISO 6579-1:2017-2 |

**Bassler, R. 1995**. Die Untersuchung von Düngemitteln-4th edition. Handbuch der landwirtschaftlichen Versuchs- und Untersuchungsmethodik (VDLUFA-Methodenbuch), vol 2.1. VDLUFA-Verlag, Darmstadt, Germany.

**(CEN) European Committee for Standardization**. **1998**. Characterization of sludge: determination of pH value (EN 12176:1998). European Committee for Standardization, Brussels, Belgium.

**(DIN) Deutsches Institut für Normung**. **2007**. Electrical laboratory devices—heating ovens and incubators (DIN 12880:2007-05). Beuth Verlag, Berlin, Germany.

**Supp. Table S2: Datasheet of the utilized potting soil (“F.-E. Typ Nullerde”, Industrie-Erdenwerk Archut GmbH & Co. KG).**

| **Components** | Bog peat (60 %), raw clay, calcium carbonate | |
| --- | --- | --- |
| **Organic Matter** | [%] | 35 |
| **pH** |  | 5.9 |
| **Salt content** | [g l^-1^ KCl] | 0.2 |
| **N** | [mg l^-1^] | 20 |
| **P_2_O_5_** | [mg l^-1^] | 10 |
| **K_2_O** | [mg l^-1^] | 40 |
| **Mg** | [mg l^-1^] | 30 |
| **S** | [mg l^-1^] | 30 |

**Supp Table S3: Test output for testing the assumptions and the significance of One-Way-ANOVA. If transformed values are given, the original data did not meet the assumption of normality.**

| **Parameter** | **p-value of**  **Shapiro-Wilk test** | **p-value of**  **Levene’s test** | **One-Way ANOVA**  **output** | **Post-hoc test** |
| --- | --- | --- | --- | --- |
| Ln(N_t_) | 0.759 | 0.067 | F(10,33) = 13.181, p<.001 | Tukey HSD |
| Ln(P_2_O_5_) | 0.438 | 0.271 | F(10,33) = 19.619, p<.001 | Tukey HSD |
| C_t_ | 0.949 | 0.000 | F(10,33) = 7.654, p<.001 | Kruskal-Wallis |
| OM | 0.123 | 0.199 | F(10,33) = 6.521, p<.001 | Tukey HSD |
| C : N ratio | 0.806 | 0.133 | F(10,33) = 10.654, p<.001 | Tukey HSD |
| Ln(N : P ratio) | 0.937 | 0.048^[[1]](https://mc.manuscriptcentral.com/jis?DOWNLOAD=TRUE&PARAMS=xik_3DN1J9UP29eP7RpqKRcHBPgDP3zAE62EL8DYkkY5qP5BQGtXAp4sc3dJfFd5NJCzJfo6zwbQocps2316YUWBqTTjzNdFrh1w3RnBaeF1A5JmzrVqnvc3KLbJZavt5Ar9itx527xixg97uY82Csa3DHeCDM138chEw2BSF6QgSzqKR1LLZ2HXMftghL4rzt731xadRiNwgrYWePtuGpJ4yq2AGy5sW4xehX1GJKf1yEeXJPARs3J2sZ6XkGVpJJMq4QGkP3L#_ftn1)^ | F(10,33) = 171.858, p<.001 | Tukey HSD |
| N : P ratio (by fertilizer)^[[2]](https://mc.manuscriptcentral.com/jis?DOWNLOAD=TRUE&PARAMS=xik_3DN1J9UP29eP7RpqKRcHBPgDP3zAE62EL8DYkkY5qP5BQGtXAp4sc3dJfFd5NJCzJfo6zwbQocps2316YUWBqTTjzNdFrh1w3RnBaeF1A5JmzrVqnvc3KLbJZavt5Ar9itx527xixg97uY82Csa3DHeCDM138chEw2BSF6QgSzqKR1LLZ2HXMftghL4rzt731xadRiNwgrYWePtuGpJ4yq2AGy5sW4xehX1GJKf1yEeXJPARs3J2sZ6XkGVpJJMq4QGkP3L#_ftn2)^ | 0.000 | 0.000 | F(4,39) = 99.218, p<.001 | Kruskal-Wallis |
| DM | 0.26 | 0.171 | F(10,33) = 3.435, p<.005 | Tukey HSD |
| Final plant height | 0.475 | 0.103 | F(10,33) = 4.849, p<.001 | Tukey HSD |
| Ln(Yield_aerial_) | 0.759 | 0.093 | F(10,33) = 7.002, p<.001 | Tukey HSD |
| Shoot : root ratio | 0.415 | 0.141 | F(10,33) = 6.439, p<.001 | Tukey HSD |
| √(Leaf area) | 0.433 | 0.134 | F(10,33) = 17.672, p<.001 | Tukey HSD |
| pH | 0.177 | 0.004 | F(10,33) = 7.348, p<.001 | Kruskal-Wallis |
| pH (by fertilizer)² | 0.090 | 0.016 | F(4,39) = 12,490, p<.001 | Kruskal-Wallis |
| Ln(N output) | 0.340 | 0.087 | F(10,33) = 71.209, p<.001 | Tukey HSD |
| N surplus | 0.142 | 0.002 | F(10,33) = 102.661, p<.001 | Kruskal-Wallis |
| Ln(NUE) | 0.340 | 0.087 | F(10,33) = 30.867, p<.001 | Tukey HSD |
| Ln(NNI) | 0.936 | 0.163 | F(10,33) = 41,167, p<.001 | Tukey HSD |

[^[1]^](https://mc.manuscriptcentral.com/jis?DOWNLOAD=TRUE&PARAMS=xik_3DN1J9UP29eP7RpqKRcHBPgDP3zAE62EL8DYkkY5qP5BQGtXAp4sc3dJfFd5NJCzJfo6zwbQocps2316YUWBqTTjzNdFrh1w3RnBaeF1A5JmzrVqnvc3KLbJZavt5Ar9itx527xixg97uY82Csa3DHeCDM138chEw2BSF6QgSzqKR1LLZ2HXMftghL4rzt731xadRiNwgrYWePtuGpJ4yq2AGy5sW4xehX1GJKf1yEeXJPARs3J2sZ6XkGVpJJMq4QGkP3L#_ftnref1) As this value was very near the level of significance and transformation should be avoided as far as possible, the assumption was considered to be met.

[^[2]^](https://mc.manuscriptcentral.com/jis?DOWNLOAD=TRUE&PARAMS=xik_3DN1J9UP29eP7RpqKRcHBPgDP3zAE62EL8DYkkY5qP5BQGtXAp4sc3dJfFd5NJCzJfo6zwbQocps2316YUWBqTTjzNdFrh1w3RnBaeF1A5JmzrVqnvc3KLbJZavt5Ar9itx527xixg97uY82Csa3DHeCDM138chEw2BSF6QgSzqKR1LLZ2HXMftghL4rzt731xadRiNwgrYWePtuGpJ4yq2AGy5sW4xehX1GJKf1yEeXJPARs3J2sZ6XkGVpJJMq4QGkP3L#_ftnref2) Depending on fertilizer type, not on the treatments
